# ﻿Ultrastructure of androconia and surrounding scales of nine species of Hesperiidae (Lepidoptera)

**DOI:** 10.3897/zookeys.1084.78883

**Published:** 2022-01-27

**Authors:** Yue Pan, Zhuoshu Yu, Xiangqun Yuan

**Affiliations:** 1 Key Laboratory of Plant Protection Resources and Pest Management, Ministry of Education, Entomological Museum, College of Plant Protection, Northwest A&F University, Yangling, 712100, China Northwest A&F University Yangling China; 2 Key Laboratory of Bio-Resource and Eco-Environment of Ministry of Education, College of Life Sciences, Sichuan University, Chengdu, 610065, China Sichuan University Chengdu China

**Keywords:** Androconia, Hesperiidae, scale, scanning electron microscope, scent glands patches

## Abstract

The ultrastructure of androconia and their surrounding scales of nine species in nine genera across four subfamilies of Hesperiidae is studied. This provides a basis for the classification and identification of some genera and species. The wing surface of the scent glands patches was cut with scissors, observed and photographed under an S-4800 scanning electron microscope (at 10.0 kV accelerated pressure). There were significant differences in the types of scent glands patches across subfamilies. The scent glands patches of Pyrginae and Dudaminae are mainly in the costal fold of the forewing, while those of Coeliadinae and Hesperiinae are mainly in the line or circular stigma on the wing surface. The length, breadth and aperture of the androconia were further measured and the data are analysed by variance and multiple comparisons. There are significant differences amongst the subfamilies, except for Dudaminae and Pyrginae. In Hesperiinae, *Telicotacolon* (Fabricius, 1775) and *Ampittiavirgata* (Leech, 1890) have no significant difference in the aperture of the androconia, but are significantly different from *Thymelicusleoninus* (Butler, 1878). There are significant differences in the aperture between *Pyrgusalveus*’s (Hübner, 1803) androconium and the second androconium of *Loboclabifasciata* (Bremer & Grey, 1853), but not with the first androconium of *Loboclabifasciata*. The morphology of androconia in the scent glands patches is very similar in Hesperiinae; all are rod-shaped and paddle-like. The scale types around the scent glands patches are different, but there are one or two similar types. To a certain extent, the aperture of the androconia reflects the genetic relationships between subfamilies and species. The differences in scale type and structure of scent glands patches can be used as a reference for the classification of subfamilies and genera in Hesperiidae.

## ﻿Introduction

Sex signs are often used as the key morphological features of Lepidoptera to distinguish males from females outdoors, such as Danaidae and Nymphalidae males possessing ear-shaped pouches on the hindwings and brush-like odour sacs at the end of their abdomen (Boppre and Vane‐Wright 1989; Chou 1998; Vane-Wright et al. 2002; Simonsen et al. 2012; de Oliveira Borges et al. 2020) or different markings on the wings of Pieridae males (Zhang 2008; Beserra Nobre et al. 2021). The male scent glands patches of Lycaenidae are marked on the abdomen dorsal plate (Ômura et al. 2015). Riodinidae scent glands patches are distributed on the wing surface, abdomen and tibia of the hind-feet of males (Hall and Harvey 2002). In Hesperiidae, the characteristics of the stigma, brand, costal fold and vein swelling on the wing surface are often used as the secondary sexual characteristics to distinguish males from females (Müller 1878; Pivnick et al. 1992; Hernández-Roldán et al. 2014; Yuan and Yuan 2015). The scent glands patches of Hesperiidae show obvious external morphological differences amongst subfamilies and genera and this has greatly attracted the attention of skipper researchers.

Previous studies have found that scent glands patches are not only obvious external morphological features, but are also closely involved in the release of pheromone. In the ultrastructure observation of the scent glands patches, it has been found that the release of pheromone is related to the special structural scales called “androconia” (Kuwahara 1979; Ômura et al. 2013; Okumura et al. 2016; Mann et al. 2017, 2020; Stamm et al. 2019). In recent years, studies of fossil scales have shown that scales had an earlier origin in the evolution of Lepidoptera and have significance in reflecting the relationships between species (Zhang et al. 2018). Many taxonomic studies, based on the morphological characteristics of the scale surface, have shown that the size, shape and surface ridges of the scales can accurately reflect the differences between species and genera. Scales have now been widely used in determining the classification, identification, evolutionary and genetic relationships of many species (Fang et al. 2007; Qiu and Han 2009).

In this paper, ultrastructural observations were made on the scales of nine representative species in four subfamilies of Hesperiidae. By comparing the types and morphological characteristics of scales that appear amongst subfamilies and genera, the differences between different taxa were analysed in order to provide a new morphological basis for studies of the classification of Hesperiidae.

## ﻿Materials and methods

### ﻿Insects

Voucher specimens representing all sampled species are deposited in the Entomological Museum of Northwest A&F University. Specimen information is presented in Table 1.

**Table 1.** Material localities and collection dates.

Subfamily	Genus	Species	Locality	Quantity
Coeliadinae	*Burara*	*B.striata*	Yifeng County, Jiangxi Province	10
	*Hasora*	*H.taminata*	Ledong County, Hainan Province	10
Dudaminae	*Lobocla*	*L.bifasciata*	Lishui City, Zhejiang Province	10
Pyrginae	*Pyrgus*	*P.alveus*	Tianshui City, Gansu Province	10
	*Erynnis*	*E.montanus*	Fuping County, Shaanxi Province	10
Hesperiinae	*Ampittia*	*A.virgata*	Nanping City, Fujian Province	10
	*Baoris*	*B.leechi*	Sanjiang City, Zhejiang Province	10
	*Thymelicus*	*T.leoninus*	Nanping City, Fujian Province	10
	*Telicota*	*T.colon*	Sanming City, Fujian Province	10

### ﻿Scanning electron microscopy

The dried wings of male skippers were selected. An appropriate size of wing surface containing the scent glands patches was cut using scissors. The samples were picked up by tweezers and were then stuck on to conductive adhesive. Each sample was given a number and its position was recorded. Samples were attached to a holder using electric adhesive tape, sputter coated with gold and observed and photographed with an S-4800 scanning electron microscope (at accelerated pressure 10.0 kV).

### ﻿Measurements and statistical analyses

Under the scanning electron microscope, the ultrastructure images of scent glands patches of nine species and their surrounding ordinary scales were obtained. Adobe Photoshop CS6 software was used to measure the length, breadth and aperture of androconia. All measurement data were analysed for variance and multiple comparisons using Excel and SPSS 24.0 software.

## ﻿Results

### ﻿Ultrastructure of androconia and surrounding scales

#### *Burarastriata* (Hewitson, 1867)

**Figure 1.**
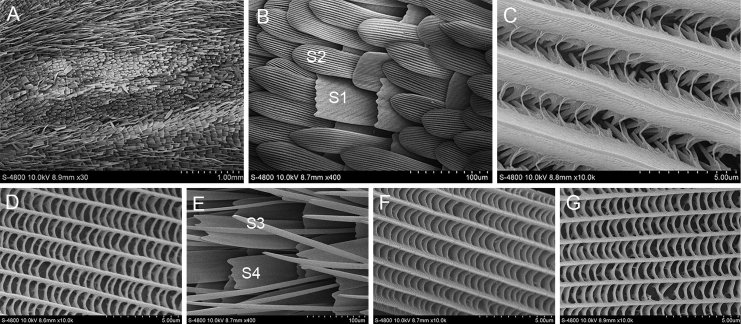
Ultrastructure of scales in and around the scent glands patches of *B.striata***A** Scent glands patches **B** Scales in the scent glands patches (S1: The first scale; S2: The second scale) **C** Ultrastructure of the first scale **D** Ultrastructure of the second scale **E** Scales around the scent glands patches (S3: The third scale; S4: The fourth scale) **F** Ultrastructure of the third scale **G** Ultrastructure of the fourth scale.

The scent glands patches of *B.striata* are marked above the 2A vein on upperside of the forewing. There are three dark brown line stigmas on both sides of the Cu1 and Cu2 veins (Fig. 1). There are two main kinds of scales. One has wavy tooth cracks at the ends and the longitudinal ridges are thick and smooth, connected by tiny transverse ribs between the longitudinal ridges. The other has blunt ends without tooth cracks and the longitudinal ridges are smooth and connected by thicker transverse ribs. Two types of scales are observed around the scent glands patches. One is a slender hairy scale and the other is a flaky scale with 3–4 teeth at the ends.

#### *Hasorataminata* (Hübner, 1818)

**Figure 2.**
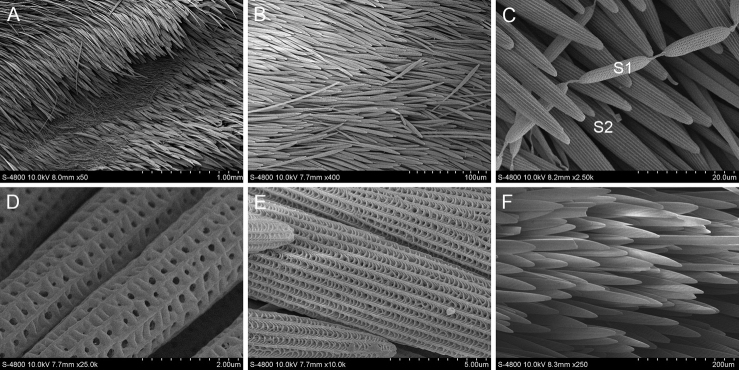
Ultrastructure of scales in and around the scent glands patches of *H.taminata***A** Scent glands patches **B** and **C** Scales in the scent glands patches (S1: The first scale (androconium); S2: The second scale) **D** Ultrastructure of the first scale **E** Ultrastructure of the second scale **F** Scales around the scent glands patches.

The scent glands patches of *H.taminata* are marked as a broken, discontinuous dark brown oval stigma on upperside of the forewing from the 2A vein to the Cu1 vein (Fig. 2). There are two kinds of scales in the scent glands patches. One is the rod-shaped androconia, which are connected by a band with constricted ends and hidden between another type of scale. The longitudinal ridges of androconia are smooth and parallel and the spacing between transverse ribs is different, forming a large number of rectangular and circular holes. The other type is neatly arranged in piles with blunt ends and narrow paddle-like scales in the middle and at the base. The longitudinal ridges of the scales are smooth and the small transverse ribs sometimes intersect similar to a pattern structure. Only one type of scales around the scent glands patches of *H.taminata* has contracted ends that are bluntly rounded.

#### *Loboclabifasciata* (Bremer & Grey, 1853)

**Figure 3.**
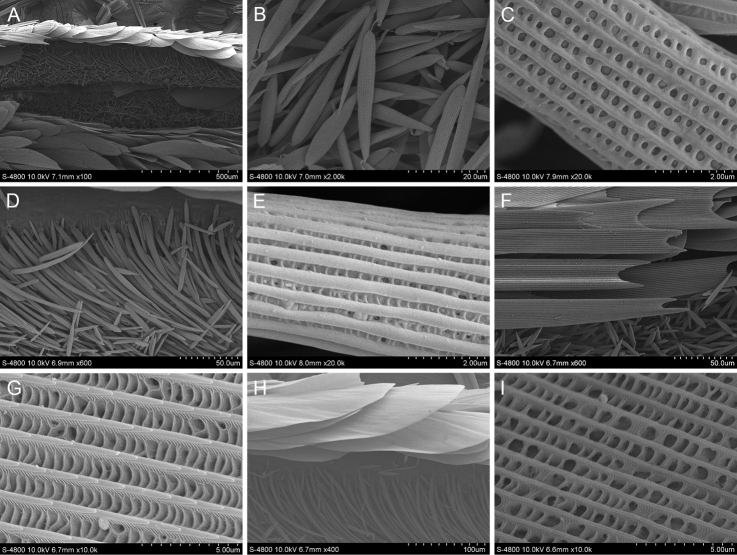
Ultrastructure of scales in and around the scent glands patches of *L.bifasciata***A** Scent glands patches **B** Type 1 scales (androconia) in the scent glands patches **C** Ultrastructure of type 1 scale in the scent glands patches **D** Type 2 scales (androconia) in the scent glands patches **E** Ultrastructure of type 2 scale in the scent glands patches **F** Type 1 scales around the scent glands patches **G** Ultrastructure of type 1 scale around the scent glands patches **H** Type 2 scales around the scent glands patches **I** Ultrastructure of type 2 scale around the scent glands patches.

The scent glands patches of *L.bifasciata* are in the costal fold on upperside of the forewing, dark brown in colour. The scent glands patches are divided into two distinct areas (Fig. 3). The lower area is scattered with short rod-shaped androconia, connected end to end in ribbons. The longitudinal ridges of the androconia are smooth and parallel. There is a row of holes with different sizes between the two longitudinal ridges and the transverse ribs are wider. The upper area has a long rod-shaped androconium with sharply contracted ends. The longitudinal ridges are connected by tiny transverse ribs, smooth without protrusions and the holes are scattered. There are two kinds of scales around the scent glands patches of *L.bifasciata*; one type has a long and narrow blade-like shape with two cleavages at the ends and another type has lateral flaky scales with broad scales and blunt ends without tooth cracks.

#### *Pyrgusalveus* (Hübner, 1803)

**Figure 4.**
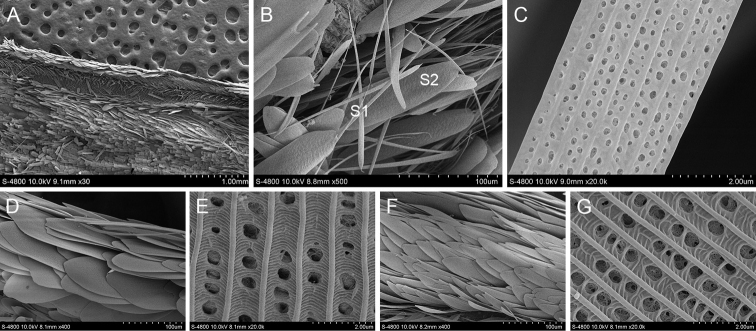
Ultrastructure of scales in and around the scent glands patches of *P.alveus***A** Scent glands patches **B** Scales in the scent glands patches (S1: The first scale (androconium); S2: The second scale) **C** Ultrastructure of the first scale in the scent glands patches **D** Type 1 scales around the scent glands patches **E** Ultrastructure of type 1 scale around the scent glands patches **F** Type 2 scales around the scent glands patches **G** Ultrastructure of type 2 scale around the scent glands patches.

The scent glands patches of *P.alveus* are marked in the costal fold on upperside of the forewing in ochre. There are mainly two kinds of scales distributed there (Fig. 4). One type is a bunch of paddle-shaped androconia. The androconia are wide at the end, narrow at the base and the longitudinal ridges are parallel. There are two rows of holes between the two longitudinal ridges. The androconia are connected by a ribbon structure. The other type is a lamellar scale with a blunt round end and no tooth cracks. There are two kinds of scales around the scent glands patches of *P.alveus*. One is a lateral flaky scale with a wide surface with blunt ends without tooth cracks and the longitudinal ridges are closely connected by flaky transverse ribs. There are holes of different sizes between the transverse ribs. The other is a long and narrow flaky scale with no or one tooth cracks at the ends. The transverse ribs have different degrees of convex ridges and the distribution density of the holes is greater than that of the other kind of scale.

#### *Erynnismontanus* (Bremer, 1861)

**Figure 5.**
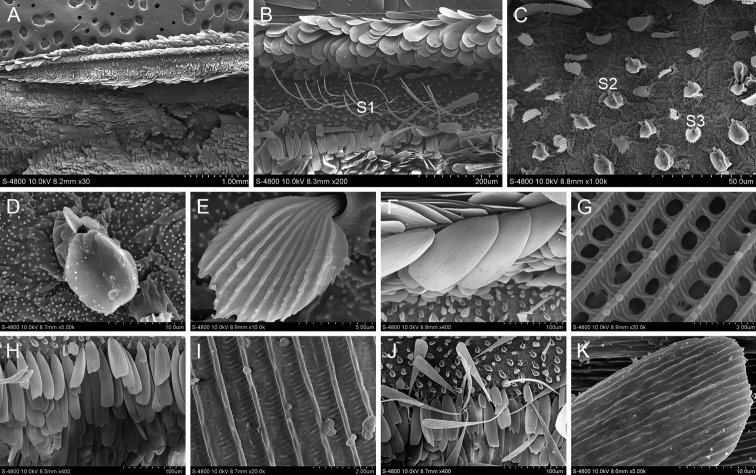
Ultrastructure of scales in and around the scent glands patches of *E.montanus***A** Scent glands patches **B** and **C** Scales in the scent glands patches (S1: The first scale; S2: The second scale; S3: The third scale) **D** The second scale **E** The third scale **F** Type 1 scales around the scent glands patches **G** Ultrastructure of type 1 scale around the scent glands patches **H** Type 2 scales around the scent glands patches **I** Ultrastructure of type 2 scale around the scent glands patches **J** Type 3 scales around the scent glands patches **K** Ultrastructure of type 3 scale around the scent glands patches.

The scent glands patches of *E.montanus* are in the costal fold on upperside of the forewing, are dark brown and composed of three kinds of scattered scales (Fig. 5). The first is a curved and upturned hairy scale around the mid-line of the scent glands patches area. The second is evenly distributed in the sex target area. The base is formed by the epidermis and there is a hole in the middle which is in the shape of a 2-leaf bud. The large leaf is like a 3-sided star and the small leaf is arc-shaped and curved. The third is young leaf-like scales with cracks. The base of the epidermis gathers in a bottle-like shape. There are three kinds of scales around the scent glands patches of *E.montanus*. The first is adjacent to the outer edge of the scent glands patches with scales standing sideways, wide on the surface and the base of the transverse ribs is wide to form a row of holes between the longitudinal ridges. The second is long and narrow, with 0, 2 or 3 teeth cracks at the ends. The connection of the transverse ribs between the longitudinal ridges is not obvious and without holes. The third is narrow at the base and round at the end, with discontinuous longitudinal ridges and no transverse rib connection between longitudinal ridges.

#### *Ampittiavirgata* (Leech, 1890)

**Figure 6.**
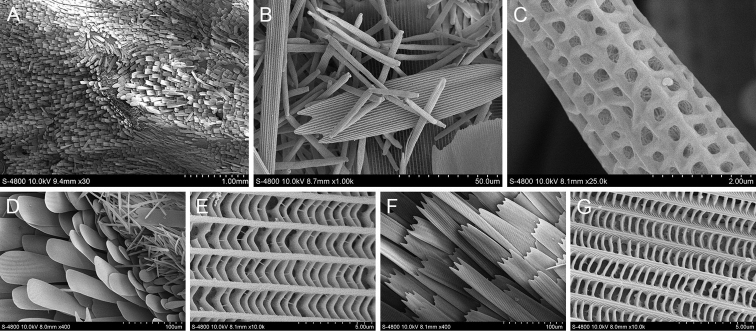
Ultrastructure of scales in and around the scent glands patches of *A.virgata***A** Scent glands patches **B** Scales (androconia) in the scent glands patches **C** Ultrastructure of the androconium **D** Type 1 scales around the scent glands patches **E** Ultrastructure of type 1 scale around the scent glands patches **F** Type 2 scales around the scent glands patches **G** Ultrastructure of type 2 scale around the scent glands patches.

The scent glands patches of *A.virgata* form a grey line stigma on upperside of the forewing from the 2A vein to the base of the Cu2 vein (Fig. 6). There is a cluster of messy rod-shaped androconia. The longitudinal ridges are nearly parallel with holes arranged in a row. The androconia are connected in pairs. There are two types of scales around the scent glands patches of *A.virgata*. One kind of scale has ends that are blunt, flat, broad and flaky. The longitudinal ridges, parallel with protrusions, are connected by the transverse ribs in the middle, which are occasionally connected by tiny filaments. The other type has 2–3 teeth clefts at the ends.

#### *Baorisleechi* (Elwes & Edwards, 1897)

**Figure 7.**
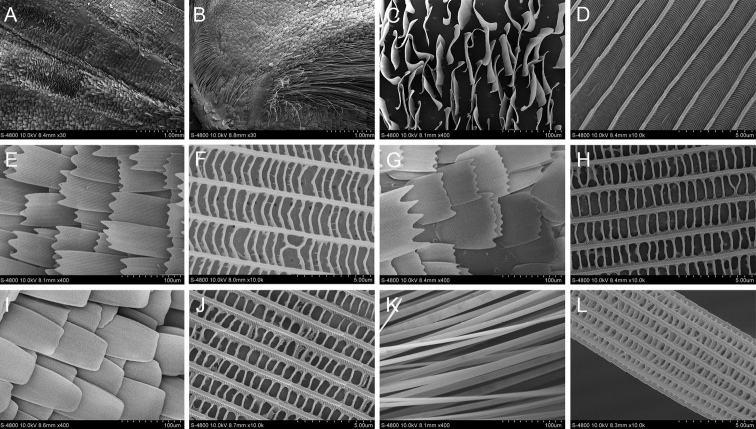
Ultrastructure of scales in and around the scent glands patches of *B.leechi***A** and **B** Scent glands patches **C** Scales in the scent glands patches **D** Ultrastructure of scale in the scent glands patches **E** Type 1 scales around the scent glands patches **F** Ultrastructure of type 1 scale around the scent glands patches **G** Type 2 scales around the scent glands patches **H** Ultrastructure of type 2 scale around the scent glands patches **I** Type 3 scales around the scent glands patches **J** Ultrastructure of type 3 scale around the scent glands patches **K** Type 4 scales around the scent glands patches **L** Ultrastructure of type 4 scale around the scent glands patches.

The scent glands patches of *B.leechi* are marked as an oval brand on underside of the forewing and two oval brands along the middle of vein 2A, dark brown in colour (Fig. 7). The scales at the brand are corrugated, neatly arranged on underside of the wing. The scales are short, erect on the wing surface, the ends are blunt and flat, the longitudinal ridges have protrusions and there are closely arranged horizontal stripes between the longitudinal ridges. Four types of scales were observed around the scent glands patches of *B.leechi*. The first has 3–4 teeth clefts at the ends. The longitudinal ridges are smooth, narrow flaky scales connected by tiny transverse ribs. The second is short with more than five teeth cracks at the ends. The structure of the transverse ribs is similar to that of the first type. The ends of the third are blunt and flat without tooth cracks and the longitudinal ridges are protruding and are connected by closely arranged transverse ribs. The fourth has longitudinal ridges with protrusions and the longitudinal ridges are connected by transverse ribs.

#### *Thymelicusleoninus* (Butler, 1878)

**Figure 8.**
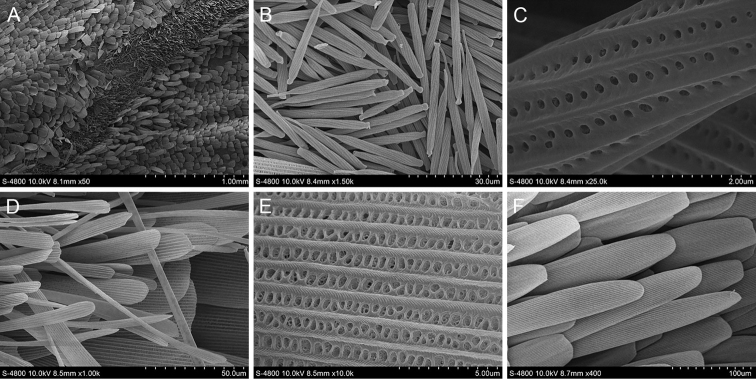
Ultrastructure of scales in and around the scent glands patches of *T.leoninus***A** Scent glands patches **B** Scales (androconia) in the scent glands patches **C** Ultrastructure of androconium **D** Type 1 scales around the scent glands patches **E** Ultrastructure of type 1 scale around the scent glands patches **F** Type 2 scales around the scent glands patches.

The scent glands patches of *T.leoninus* are oblique black line stigmas from the 2A vein to the base of the Cu1 vein on upperside of the forewing. There are two kinds of scales distributed there (Fig. 8). The first is disorderly arranged rod-shaped androconia. The longitudinal ridges of the androconia are left helixes. The longitudinal ridges are connected by wider transverse ribs to form rows of holes. The second kind are paddle-like scales with smooth longitudinal ridges and holes between the longitudinal ridges. These holes vary in size, sometimes leaning to one side of the longitudinal ridge. There is one kind of scale around the scent glands patches of *T.leoninus*, long and narrow flaky scales with blunt ends.

#### *Telicotacolon* (Fabricius, 1775)

**Figure 9.**
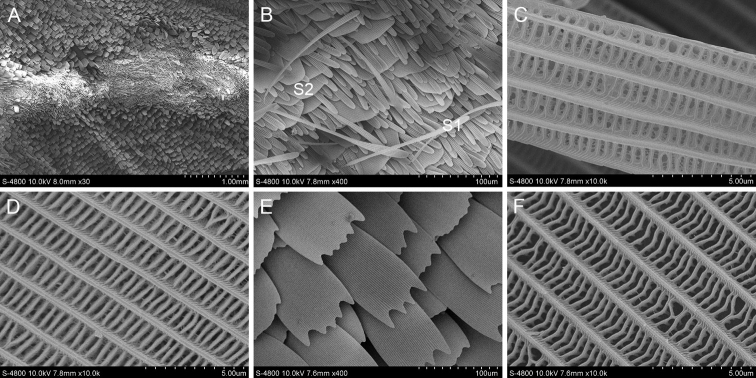
Ultrastructure of scales in and around the scent glands patches of *T.colon***A** Scent glands patches **B** Scales in the scent glands patches (S1: The first scale (androconium); S2: The second scale) **C** Ultrastructure of the first scale **D** Ultrastructure of the second scale **E** Scales around the scent glands patches **F** Ultrastructure of the scale around the scent glands patches.

*T.colon* has a grey line stigma marked in the area medialis of upperside of the forewing. There are two kinds of scales distributed there (Fig. 9). The first are paddle-shaped androconia. The longitudinal ridges at the ends of the scales are connected by tiny transverse ribs. From the end to the middle of these scales, the rows of holes between the ribs gradually change from horizontal strips to round holes, resembling a rod-shaped androconial structure. The second has blunt ends without tooth cracks, wide and flaky scales, smooth longitudinal ridges and rows of transverse ribs between the longitudinal ridges and with some transverse ribs intersecting at the base. There is one type of scale around the scent glands patches of *T.colon*. The flaky scales have 4–6 teeth cracks at the ends. The longitudinal ridges are smooth and parallel without protrusions. They are connected by transverse ribs and some transverse ribs intersect in the middle.

### ﻿Androconia

Androconia were observed in seven of the nine species selected. The length, breadth, aperture, shape and longitudinal ridge direction of the androconia were observed and measured (Table 2). The androconia have been analysed by variance analysis amongst species and subfamilies.

**Table 2.** The length, breadth, aperture, shape, longitudinal direction, number and multiple comparisons of the seven androconia.

	Coeliadinae	Dudaminae		Pyrginae	Hesperiinae		
	*H.taminata*	*L.bifasciata* I	*L.bifasciata* II	*P.alveus*	*A.virgata*	*T.leoninus*	*T.colon*
Length/μm	160.886 ± 17.517 (Aa)	17.339 ± 0.728 (Dd)	68.802 ± 3.502 (Bb)	154.427 ± 6.985 (Aa)	35.890 ± 1.172 (Cc)	29.377 ± 1.319 (CDc)	172.400 ± 5.208 (Aa)
Number	33	56	18	21	66	92	32
Breadth/μm	2.459 ± 0.100 (Dd)	2.928 ± 0.101 (CDc)	2.841 ± 0.094 (CDcd)	4.326 ± 0.211 (Bb)	3.195 ± 0.082 (Cc)	2.944 ± 0.039 (Cc)	7.923 ± 0.128 (Aa)
Number	44	43	40	59	73	96	94
Aperture/μm	0.200 ± 0.006 (Bb)	0.166 ± 0.004 (CDc)	0.140 ± 0.008 (Dd)	0.172 ± 0.006 (Cc)	0.318 ± 0.006 (Aa)	0.204 ± 0.004 (Bb)	0.323 ± 0.008 (Aa)
Number	108	71	39	104	145	123	70
Shape	rod	rod	rod	paddle	rod	rod	paddle
Longitudinal ridge direction	parallel	parallel	parallel	parallel	parallel	left helix	parallel
Length/μm	160.886 ± 17.517 (Aa)	29.857 ± 2.770 (Cc)		154.427 ± 6.985 (Aa)	55.727 ± 3.994 (Bb)		
Number	33	190		21	190		
Breadth/μm	2.490 ± 0.096 (Bb)	2.935 ± 0.085 (Bb)		4.521 ± 0.233 (Aa)	4.755 ± 0.154 (Aa)		
Number	47	83		59	260		
Aperture/μm	0.200 ± 0.006 (Bb)	0.157 ± 0.004 (Cc)		0.172 ± 0.006 (Cc)	0.277 ± 0.005 (Aa)		
Number	108	110		104	338		

Each value retains 3 significant digits. Data are presented as Mean ± SE; Number: sample size. Different capital letters on the same line indicate extremely significant differences (α = 0.01). Different lower case letters on the same line indicate extremely significant differences (α = 0.05).

In terms of length, the results of multiple comparisons of androconia show that there are significant differences amongst the subfamilies, except for Coeliadinae and Pyrginae. Except for the group of *T.colon*, *H.taminata* and *P.alveus* and another group of *A.virgata* and *T.leoninus*, there were significant differences amongst species (α = 0.05).

In terms of breadth, the results of comparing androconia show that there are significant differences amongst the subfamilies, except for Hesperiinae and Pyrginae, Coeliadinae and Dudaminae. There are no significant differences in the breadth of the two androconia of *L.bifasciata*, *A.virgata* and *T.leoninus*, *L.bifasciata* and *H.taminata*, but there are significant differences amongst species (α = 0.05).

In terms of aperture, the results of androconia comparisons show that there are significant differences amongst the subfamilies, except for Dudaminae and Pyrginae. There are no significant differences in the aperture of androconia of *T.colon* and *A.virgata*, *H.taminata* and *T.leoninus*, *L.bifasciata* I and *P.alveus*, but there are significant differences amongst species (α = 0.05).

## ﻿Discussion

### ﻿Scent organs

There are three main locations for scent organs on butterflies: a) Wings. There are different manifestations in different families of Lepidoptera. For example, the main scent glands patches of Danainae and Heliconiinae are in a small part of the central forewing and on the hindwing. Their location on the hindwing is obvious amongst different genera. In addition, there is a specialised bag-like structure on the ventral surface of the hindwing (Boppre and Vane‐Wright 1989; Vane-Wright et al. 2002; de Oliveira Borges et al. 2020). In Pieridae, the scent glands patches are mainly concentrated in the area where the forewings and hindwings overlap (Beserra Nobre et al. 2021). The scent glands patches of Riodinidae and Lycaenidae are located at the forewing tip (Hall and Harvey 2002; Ômura et al. 2015). b) Abdomen. These are characterised by specific plaques distributed in different areas of the abdomen and in tufts of hair at the end of the abdomen that can be turned out in Danaidae and Riodinidae (Hall and Harvey 2002; Simonsen et al. 2012; de Oliveira Borges et al. 2020). c) Appendages. This mainly refers to the upright hair tufts or hair pencils on the hind tibia, which have been reported in Riodinidae, *Pyrgus* and *Coladenia* in Hesperiidae (Hall and Harvey 2002; Hernández-Roldán et al. 2014).

The scent organs of Hesperiidae are mainly concentrated in the first type, especially the black, smooth and raised scars on upperside of the forewing, such as a brand formed after animal skin burns, with even a distortion of the veins where they are located. There are obvious differences in markings on the wing surface of other families (Yuan and Yuan 2015).

### ﻿Scales

Since people first became aware of butterflies, they have attracted the attention of researchers with their colourful appearance. The significance of the unique scales of butterflies lies not only in the appreciation of the external image of the butterfly, but also continues to promote the development of bionic technology, biogeography, paleontology and other fields (Ghiradella 1991; Zhong and Shen 2003; Han et al. 2008; Simonsen et al. 2012; Zhang et al. 2015; Yang and Chen 2021). Butterfly scales are divided into basal scales and cover scales. The basal scales are located on the surface of the wing’s membrane and the cover scales cover the basal scales. Most of the basal scales have two to five cracks at the ends, while the brightly coloured surface scales without cracks are smooth, straight or curved (Fang et al. 2007; Han et al. 2008; Qiu and Han 2009; Simonsen et al. 2012; Parnell et al. 2018). The surrounding scales of the nine species of Hesperiidae are composed of more than two kinds of scales, except for *T.colon*, which has only one basal scale. *B.striata*, *H.taminata*, *L.bifasciata*, *P.alveus*, *A.virgata* and *T.leoninus* have one basal scale and one cover scale; *E.montanus* has one basal scale and two cover scales; and *B.leechi* has two basal scales and two cover scales. The scales of scent glands patches showed one type of androconia and one type of cover scale of *H.taminata*; two kinds of androconia with different shapes and different distributions of *L.bifasciata*; one kind of paddle-shaped androconia and one kind of cover scale of *P.alveus* and *T.colon*; *T.leoninus* with one kind of androconia and one kind of paddle-shaped cover scale; and *A.virgata* with one kind of paddle-shaped androconia. No androconia have been found in *B.striata*, *E.montanus* and *B.leechi*. *B.striata* has two kinds of ordinary scales: one type of basal scale and one kind of cover scale. *E.montanus* has three kinds of ordinary scales: one kind of basal scale (tender leaf with tooth cracks) and two kinds of cover scales (hairy and bud-like). *B.leechi* has one kind of cover scale (tile-like).

The scales of Nymphalidae have similar shapes, structures and arrangements, especially the shape and size of the ultrastructure of the wing scales of the same genus which are small, indicating that the genetic relationship between them is close (Fang et al. 2007; Darragh et al. 2017; Stamm et al. 2019). Amongst the four subfamilies observed, *P.alveus* and *E.montanus* of Pyrginae; *A.virgata*, *B.leechi*, *T.leoninus* and *T.colon* of Hesperiinae; and Dudaminae and Pyrginae have one to two species with extremely similar scale types around the scent glands patches. However, this feature has not been found in *B.striata* and *H.taminata* of Coeliadinae.

### ﻿Androconia

In butterfly behavioural experiments, some studies have shown that the pheromone released from the scent organs plays a decisive role in the identification of related species (Andersson et al. 2007; Friberg et al. 2008; Ômura et al. 2013; Darragh et al. 2019). Previous studies have shown that the androconia are special glandular scales which are the main structural components of the male courtship pheromone system in Lepidoptera (Pivnick et al. 1992; Hall and Harvey 2002; Beserra Nobre et al. 2021).

The morphology of androconia is significantly distinctive amongst different families in Lepidoptera. For example, androconia are oval and flaky in Pieridae, fan-shaped in Lycaenidae, coronal-shaped in Nymphalidae and rod-shaped or paddle-shaped in Hesperiidae. It can be seen that the morphology of androconia can be used as an obvious morphological characteristic for family classification (Kuwahara 1979; Pivnick et al. 1992; Vane-Wright et al. 2002; Ômura et al. 2015; Darragh et al. 2017; Beserra Nobre et al. 2021). A study showed that the morphology of androconia in the same genus can be different, although 13 species of *Celastrina* (except *C.ladon*) have fan-shaped androconia. In this study, some species had differences in the size, lamellar microstructure and number of longitudinal ridges of androconia. In addition, the three subspecies of *C.argiolus* showed significant changes in androconia morphology (Ômura et al. 2015). In the study of the ultrastructure of androconia in Coliadinae, it was seen that the distribution density of androconia is significantly larger than that of the surrounding ordinary scales. This phenomenon is very obvious in Hesperiidae, which seems to alleviate the problem of insufficient wing surfaces. It is speculated that this morphological feature is closely related to the release of a pheromone (Pivnick et al. 1992; Beserra Nobre et al. 2021).

Analysing the observed morphological characteristics of the seven species of androconia in Hesperiidae, it is found that the aperture of androconia is the largest in Hesperiinae. Amongst them, the mean value for *T.colon* is 0.323 μm, *A.virgata* is 0.318 μm and *T.leoninus* is 0.204 μm, followed by *H.taminata* at 0.200 μm in Coeliadinae and *P.alveus* at 0.172 μm in Pyrginae. The mean values of apertures are 0.166 μm and 0.140 μm in *L.bifasciata*. Through multiple comparative analyses, the apertures were found to be extremely different amongst species and subfamilies and the lengths and breadths of androconia were extremely different amongst subfamilies. The classification analysis of apertures is more consistent with existing domestic research in Hesperiidae: (Coeliadinae + (Pyrginae + (Dudaminae + (Heteropterinae + Hesperiinae)))) (Warren et al. 2008, 2009; Yuan 2010). The left helixes of the longitudinal ridge seem to be a unique feature. Is it unique to *T.leoninus*, to the genus *Thymelicus* or is it a feature possessed by a higher category? It can, thus, be seen that it is very important to extract the morphological characteristics of androconia insofar as it is of fundamental significance in reflecting our understanding of the phylogenetic relationships between species.

## ﻿Conclusions

Based on the above observation results, it is proposed that the types of scales around the scent glands patches, the presence of androconia in the scent glands patches and their types and morphological characteristics can be used as the basis for classification of different genera and species within the subfamily. Under further variance analysis and multiple comparisons of the length, breadth and aperture data of seven kinds of androconia, it is found that the data on androconial apertures fit the existing classification system better than the data on their length and breadth. This provides further knowledge of significance for phylogenetic research on Hesperiidae.
